# Physics-Guided Neural Phase Demodulation for Non-Contact Laser-Ultrasound Sensing of Corneal Phantom Deformation

**DOI:** 10.3390/bios16100577

**Published:** 2026-10-13

**Authors:** Muhammad Awais, Younggue Kim, Byeong Ha Lee

**Affiliations:** 1 Department of Electrical Engineering and Computer Science, Gwangju Institute of Science and Technology, Gwangju 61005, Republic of Korea; 2 Korea Photonics Technology Institute, Gwangju 61007, Republic of Korea

**Keywords:** non-contact acoustic detection, optical ultrasound detection, 3 × 3 fiber-optic interferometer, photoacoustic detection, laser ultrasound (LUS), physics-guided deep learning, corneal deformation sensing

## Abstract

Non-contact detection of weak, transient acoustic signals is essential for photoacoustic and laser-ultrasound (LUS) bioimaging, including the assessment of corneal deformation and thickness variation. Passive 3 × 3 fiber-coupler interferometers provide phase-diverse sensing without external modulation; however, conventional ellipse-based calibration becomes unreliable when the measured phase excursion covers only a small fraction of the characteristic ellipse, as in nanometer-scale corneal responses. This study presents a physics-guided temporal convolutional network (PG-TCN) that directly maps two nonideal interferometric outputs to a continuous dynamic phase signal without ellipse fitting or separate phase unwrapping during inference. The model combines dilated one-dimensional convolutions with interferometric-consistency and temporal-regularization losses. Tests using PZT-modulated calibration signals acquired under several static chamber pressures of 10, 15, 20, and 25 kPa showed lower mean absolute error, higher normalized cross-correlation, and lower displacement RMSE than a CNN-LSTM baseline. In corneal-phantom LUS measurements, the first detected echo shifted from 4.632 to 4.488 µs between 10 and 20 kPa, corresponding to a relative thickness reduction of approximately 7.8 µm/kPa. The PG-TCN required 210 ± 18 µs per sequence while using approximately half the trainable parameters of the CNN-LSTM baseline. These results demonstrate a fast physics-guided optical detection framework for non-contact sensing of weak acoustic responses, supporting the use of 3 × 3 optical interferometry as a biosensing approach for LUS and photoacoustic applications.

## 1. Introduction

Photoacoustic imaging and laser-ultrasound (LUS) sensing use pulsed optical excitation to generate acoustic responses that provide structural, functional, or mechanical information about biological specimens [1,2,3,4]. Reliable measurement of these broadband and transient acoustic signals depends strongly on the sensitivity, stability, and temporal resolution of the detection system. Advanced detection and signal-processing methods are therefore important for converting weak acoustic responses into quantitative measurements, particularly for delicate or mechanically sensitive biological surfaces.

In this study, the main work focuses specifically on the detection stage rather than on ultrasound image formation. Optical interferometric sensing provides a non-contact alternative to piezoelectric detection by converting the acoustically induced surface motion into optical phase variations. Such photonic detection is directly relevant to photoacoustic and LUS systems in which weak and transient acoustic waves must be measured without mechanically contacting the specimen.

Many conventional photoacoustic and LUS systems employ piezoelectric transducers (PZTs) that require physical contact or an acoustic coupling medium. Although these detectors are well established, contact may perturb sensitive surfaces, introduce coupling-dependent variability, and reduce measurement repeatability. These limitations are particularly relevant to ophthalmic applications because the cornea is transparent, curved, and mechanically deformable. Corneal thickness and deformation are associated with intraocular pressure, hydration, biomechanical integrity, glaucoma assessment, keratoconus screening, and the interpretation of tonometry measurements [5,6,7,8,9]. Non-contact detection of LUS-induced surface motion therefore provides a useful experimental approach for investigating corneal-phantom deformation and thickness-related responses without mechanically loading the sample.

Optical interferometry is well suited to this purpose because nanometer-scale surface displacement can be converted into measurable optical phase variations without making direct mechanical contact [10,11,12]. Fiber-optic interferometers offer additional advantages, including compactness, high displacement sensitivity, immunity to electromagnetic interference, and compatibility with remote sensing configurations [13,14]. In particular, interferometers based on 3 × 3 fiber couplers provide passive phase-diverse outputs with relative phase offsets of approximately 2π/3 [15,16,17,18,19,20,21]. Other dual-channel quadrature schemes can also provide phase diversity, typically through an imposed phase shift or additional optical components. However, the 3 × 3 coupler provides intrinsic phase diversity within a passive and compact all-fiber configuration, avoiding the need for external phase modulation or active operating-point stabilization. Although three outputs of the interferometer are available, any two outputs contain sufficient relative phase information, after calibration, to define a two-dimensional Lissajous trajectory and recover the interferometric phase. The present implementation therefore detects two outputs, consistent with our previously established system [22], reducing the required number of photodetectors and digitizers. The interferometer therefore functions as a non-contact optical acoustic-wave sensor that converts the acoustically induced weak surface displacement into phase-diverse optical intensity signals.

Accurate phase recovery nevertheless remains difficult under practical operating conditions. Unequal coupler splitting ratios, detector-gain mismatch, DC offsets, nonideal intrinsic phase differences, polarization fluctuations, variations in sample reflectivity, and environmental drift can distort the measured intensity signals. Conventional methods based on arctangent reconstruction or ellipse fitting generally require calibration, Lissajous-trajectory estimation, and phase unwrapping [15,16,17,22]. Their performance deteriorates when the phase excursion is small and the measured trajectory represents only a short arc of the characteristic ellipse. This condition is common in LUS measurements, where surface displacements may be only a few nanometers. For a 1550 nm probe in a reflection interferometer, a complete 2π phase cycle corresponds to 775 nm of surface displacement; consequently, nanometer-scale displacement in the surface of a sample makes only a small fraction of the characteristic ellipse. Ellipse-parameter estimation can therefore become ill-conditioned and highly sensitive to noise.

In our previous work, a 3 × 3 fiber-coupler interferometer was used to measure nanometer-scale LUS-induced displacement and estimate the thickness of a corneal phantom [22]. Repeated acquisitions enabled reconstruction of the characteristic ellipse, while coherent averaging reduced the noise-equivalent displacement. However, changes in the chamber pressure modified the phantom curvature and, consequently, the optical response, requiring the ellipse parameters to be re-estimated for each pressure condition. Although effective, this procedure increases acquisition and processing requirements and remains sensitive to variations in the interferometric operating conditions. The present study addresses this phase-demodulation limitation while retaining the established non-contact optical sensing configuration.

Deep learning offers an alternative for phase recovery by learning nonlinear mappings between interferometric signals and phase sequences. Neural networks have been applied to phase unwrapping, phase denoising, ultrasonic signal processing, and physics-consistent reconstruction [23,24,25,26,27,28]. However, most previous studies focus on two-dimensional phase data, whereas fiber-optic LUS produces one-dimensional temporal signals. Therefore, temporal convolutional networks efficiently capture multiscale temporal features [29,30], while physics-based constraints can promote physically consistent phase predictions [31,32].

In this work, we propose a physics-guided temporal convolutional network (PG-TCN) for direct phase demodulation from two measured outputs of a 3 × 3 fiber-optic interferometer. The network estimates a continuous optical phase sequence directly from the two measured output channels. During inference, it does not require explicit ellipse fitting, arctangent inversion, quadrature reconstruction, or a separate phase-unwrapping procedure. Dilated temporal convolutions capture multiscale temporal features, while interferometric-consistency and temporal-regularization terms promote agreement with the measured optical response and suppress nonphysical phase fluctuations.

The proposed method is evaluated using PZT-modulated calibration measurements and LUS echo signals obtained from a cornea-mimicking phantom. Its performance is compared with conventional ellipse-fitting demodulation and a CNN-LSTM neural demodulator [33]. The main contributions of this work are as follows:

- A physics-guided temporal convolutional network that directly recovers continuous dynamic phase from two interferometric intensity sequences without explicit ellipse fitting or separate phase unwrapping during inference.
- A computationally efficient phase-demodulation framework that uses approximately half the trainable parameters of the CNN-LSTM baseline while providing lower inference latency and improved displacement-recovery accuracy.
- Experimental validation of the proposed framework by using PZT-modulated calibration and a corneal-mimicking phantom, including displacement recovery and relative thickness-change estimation from acoustic echo arrival times.

The results demonstrate the potential of physics-guided phase demodulation as an enabling signal-processing component for non-contact photonic detection of weak acoustic responses. The experimental validation is made by using the LUS-induced surface deformation with a corneal phantom.

## 2. Materials and Methods

### 2.1. Non-Contact Optical Detection of LUS Signals

The experimental platform was a reflection-type Mach-Zehnder interferometer based on a 3 × 3 fiber coupler. The interferometer therefore serves as a non-contact optical ultrasound detector: LUS-induced surface motion modulates the optical phase of the sensing arm, and the resulting phase variation is recovered from two outputs of the multi-channel interferometer. The interferometer configuration, acquisition hardware, and corneal-phantom fabrication procedure were reported in detail in our previous study [22]; here, only the elements essential to the present phase-demodulation approach are summarized. In the previous work, the two detected interferometric signals were processed using conventional ellipse-fitting-based phase recovery, whereas the present study employs a PG-TCN demodulator for direct phase demodulation.

The cornea-mimicking phantom consisted of a carbon-black-doped PDMS film bonded to a PDMS block containing an air chamber. The film thickness was approximately 2–2.5 mm, and the chamber pressure was controlled using a syringe pump [22]. The phantom was designed as a deformable pressure-dependent membrane model rather than as a complete anatomical or optical replica of the human cornea. Increasing chamber pressure changes the curvature and thickness of the PDMS film, thereby providing a controlled model for evaluating pressure-dependent surface deformation and acoustic propagation time. Carbon black was incorporated into the PDMS to increase absorption of the 532 nm excitation light pulse and thereby facilitate generation of the LUS pulse near the upper film surface.

The LUS excitation was provided by a 532 nm Nd:YAG laser (Brilliant Ultra 50, Quantel, Cournon-d’Auvergne, France) operated at a repetition rate of 20 Hz. The excitation pulse was delivered through a multimode fiber and collimated onto the phantom with an approximate beam diameter of 5 mm [22]. The generated LUS pulse propagated through the PDMS film and was reflected at the lower PDMS–air interface, which provides a strong acoustic boundary because of the large difference in the acoustic impedance. The reflected LUS pulse returned to the upper surface and produced the transient surface displacement, which was finally measured by the interferometer. In the longer acquisition used in Ref. [22], repeated reflections between the upper film surface and the PDMS–air interface produced multiple echo responses, whereas the shorter acquisition window used in the present study captured only the first reliable echo. During the LUS measurements, reference-arm PZT modulation was disabled, and the time-dependent interferometric response originated from the LUS-induced surface displacement was utilized.

The interferometric probe used a 1550 nm continuous-wave laser (SFL1550P, Thorlabs, Newton, NJ, USA). In the experimental configuration, the probe beam was split in a 99:1 ratio between the sample and reference arms [22]. Optical circulators placed in each arm directed the reflected light from the reference mirror and the phantom surface, respectively, to a 3 × 3 fiber coupler, where the two fields were recombined. Two outputs of the coupler were simultaneously detected by two photodetectors and digitized at 125 MS/s, yielding the temporal intensity signals I_1_(t) and I_2_(t). The excitation pulse duration, pulse energy or fluence at the phantom, probe-beam power, and probe spot size were not available in the archived experimental information and therefore could not be specified retrospectively. Although the 3 × 3 coupler provides three output ports, the present system uses two simultaneously detected outputs, consistent with the previously established experimental configuration [22]. Because any two outputs have a nonzero relative phase separation, their joint response provides the two-dimensional phase-diverse information required for ellipse-based calibration and neural phase demodulation. The third output was therefore not required for the present implementation, although its inclusion could provide additional redundancy under degraded signal conditions. The two-channel configuration also reduces the hardware and acquisition requirements of the detection system by requiring only two photodetectors and two digitizer channels. Each simultaneously acquired channel pair provides a 2 × 1024 temporal input to the neural demodulator, allowing continuous phase to be recovered directly without additional interferometric measurements during inference. This configuration therefore provides a practical balance between the phase-diverse optical detection and the reduced acquisition complexity.

All interferometric datasets analyzed in this study were experimentally acquired using the physically implemented 3 × 3 fiber-optic interferometric system; no simulated interferometric signals were used. The experimental platform and measurement procedure are also described in Ref. [22]. Each raw interferometric channel was independently min–max normalized on a sequence-by-sequence basis to the range [0, 1], using the minimum and maximum values of that individual channel record; fixed calibration-derived normalization limits were not used. The resulting normalized channel pair was then supplied to the PG-TCN as its two-channel input. Because normalization is applied independently to each measured channel, multiplicative variations in the detected optical intensity arising from source-power fluctuations or uniform transmission loss are largely removed before neural inference. However, normalization does not compensate for the reduction in signal-to-noise ratio that can accompany lower received optical power.

**Figure 1:**
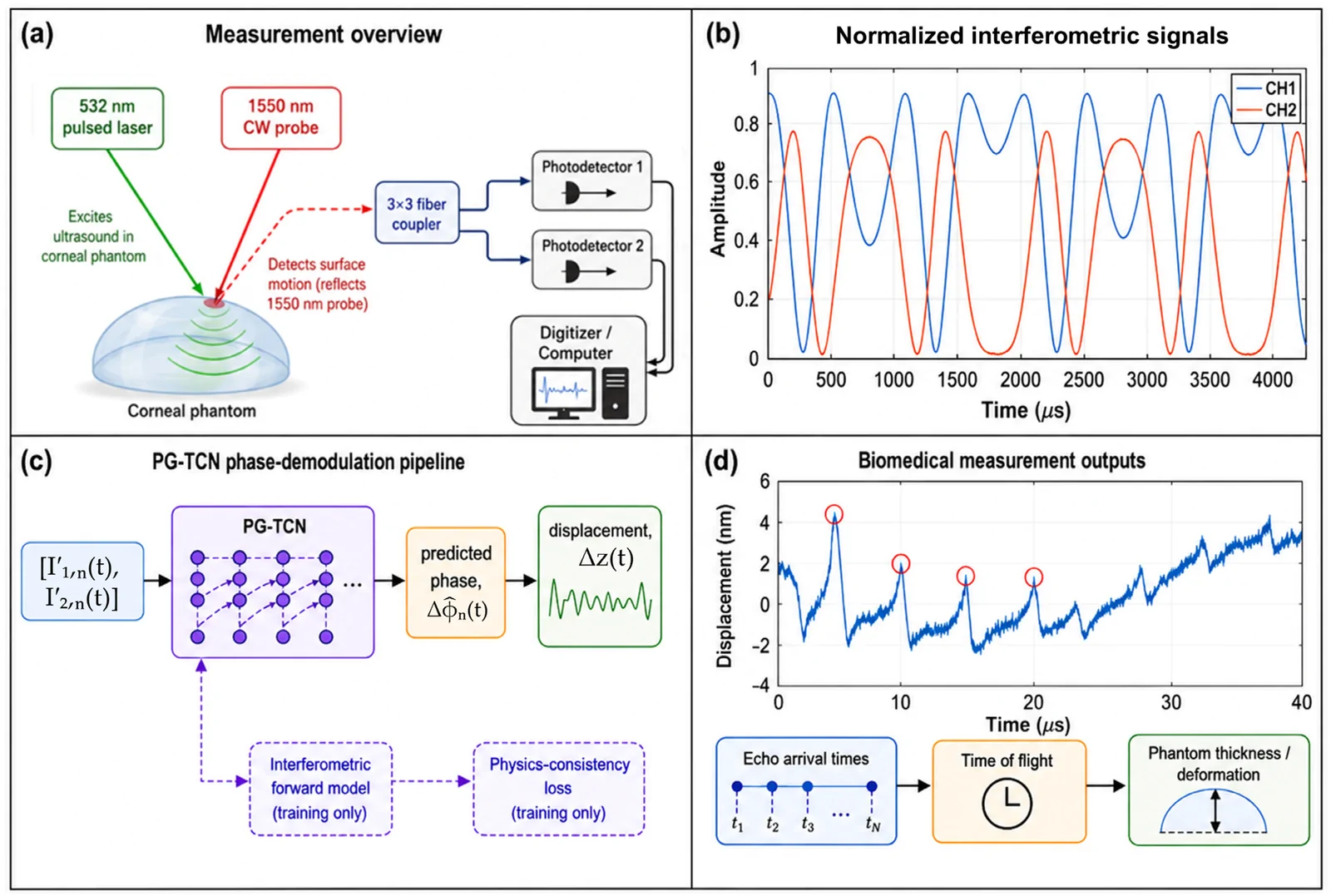
Overview of the non-contact laser-ultrasound sensing and PG-TCN demodulation workflow. (**a**) Schematic of the physically implemented non-contact LUS measurement system. (**b**) Representative normalized interferometric signals; (**c**) PG-TCN processing pipeline, in which the two normalized intensity sequences are used to predict the dynamic phase, while the interferometric forward model and physics-consistency loss are applied during training. (**d**) Recovered displacement signal and time-of-flight analysis for corneal-phantom thickness and deformation assessment. Red circles are detected peaks.

After phase recovery, the dynamic phase change Δϕ(t) was converted into surface displacement Δz(t) using the reflection-mode interferometric relation

(1) $$\Delta z(t)=\frac{\Delta \phi (t)}{2k_{0}}=\frac{\lambda \Delta \phi \left(t\right)}{4\pi }$$

where k_0_ = 2π/λ is the optical wavenumber, and λ = 1550 nm is the probe wavelength. The recovered displacement waveform was then analyzed to identify the LUS-induced echo responses and determine the time-of-flight (TOF) of the acoustic pulse. Figure 1 provides an overview of the non-contact LUS sensing and PG-TCN demodulation workflow. Figure 1a illustrates the simplified optical measurement configuration, in which the 532 nm pulsed laser generates ultrasound pulses and the 1550 nm probe detects the resulting surface motion or displacement. Figure 1b shows representative two-channel interferometric signals recorded produced with a slowly varying motion in the reference arm. As shown in Figure 1c, these two signals are supplied to the PG-TCN to recover the dynamic phase and displacement, while the interferometric forward model and physics-consistency loss are used only during training. Figure 1d shows the recovered surface displacement induced by the LUS pulse obtained with a corneal-phantom thickness. The subsequent identification of acoustic echo arrival times allows us to have the TOF-based assessment of corneal-phantom thickness and deformation.

### 2.2. Physics-Guided Temporal Convolutional Network

**Figure 2:**
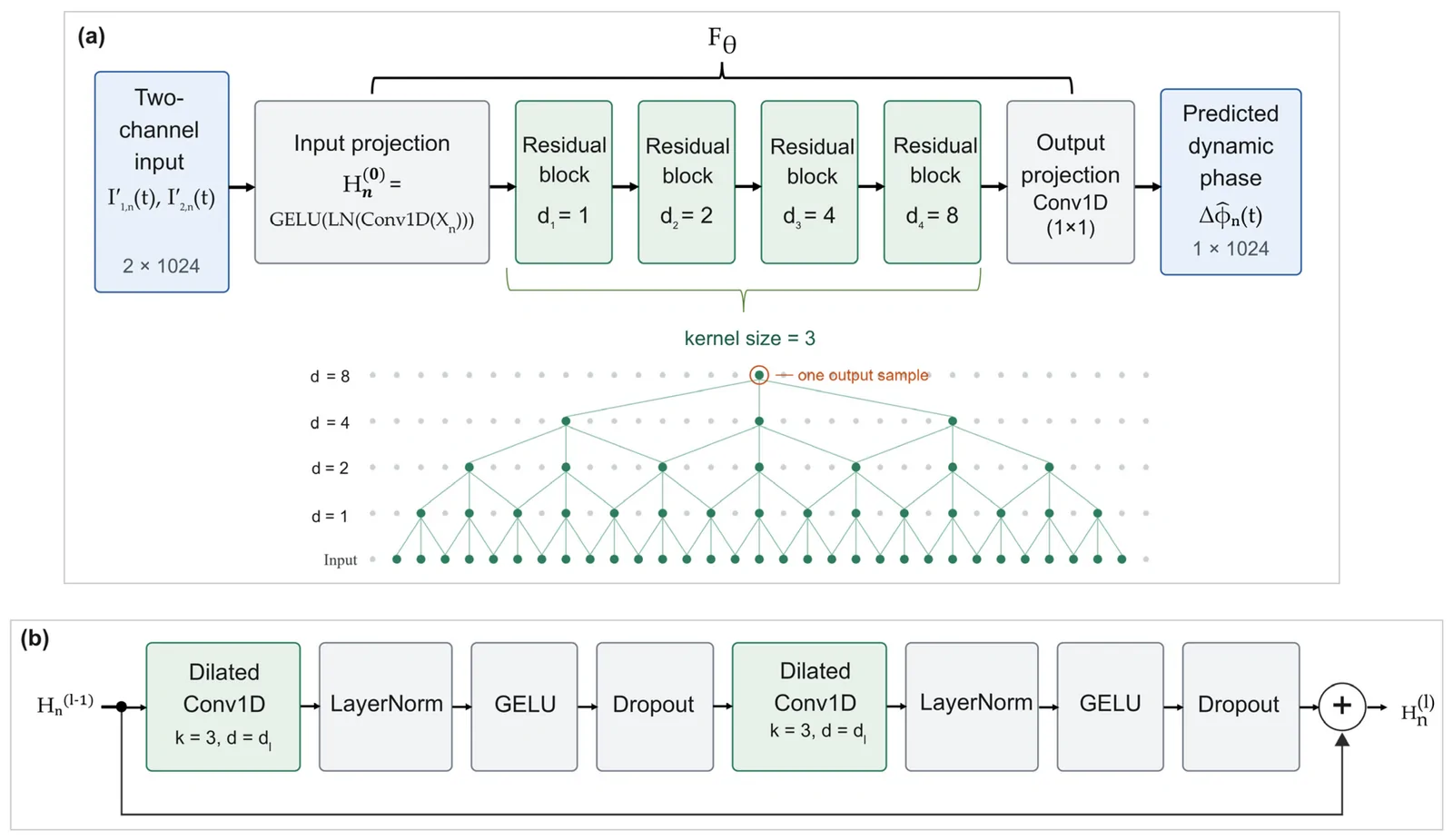
Architecture of the proposed physics-guided temporal convolutional network (PG-TCN). (**a**) Overall network structure, comprising two-channel normalized interferometric input, pointwise input projection, four residual dilated temporal blocks with dilation factors of 1, 2, 4, and 8, a 1 × 1 output projection, and prediction of the dynamic-phase sequence Δ$\hat{\phi }$_n_(t). The lower diagram illustrates the progressive expansion of temporal context through the dilated convolutional blocks. (**b**) Structure of an individual residual temporal block containing two non-causal dilated Conv1D–Layer Norm–GELU–Dropout stages and an identity shortcut connection.

The proposed PG-TCN recovers the continuous dynamic optical phase directly from the two normalized intensity signals measured by the 3 × 3 interferometer. The network architecture is illustrated in Figure 2: panel (a) presents the overall PG-TCN structure, while panel (b) shows the configuration of a residual temporal block. Each network input contained two synchronized intensity sequences with T = 2024 temporal samples, normalized as described in Section 2.1. The corresponding reference phase sequence was not normalized and remained expressed in radians. The normalization preserves the normalized temporal waveform shape and interchannel phase relationships used for phase demodulation, while the original absolute modulation amplitude is not retained. The two-channel input at time “t” was represented as

X_n_(t) = [I′_1_,_n_(t), I′_2_,_n_(t)]^T^, t = 1, 2, …, T (2)

where X_n_(t) is the n-th normalized input sequence, and I′_1_,_n_(t) and I′_2_,_n_(t) are the normalized interferometric signals. The PG-TCN maps this sequence to a continuous dynamic-phase sequence according to

(3) $$\Delta {\hat{\phi }}_{n}(t)=F_{\theta }[X_{n}]$$

where F_θ_ denotes the PG-TCN with trainable parameters θ, and Δ$\hat{\phi }$_n_(t) is the predicted dynamic phase change. Each input had a dimension of 2 × 1024, while the corresponding output phase sequence had a dimension of 1 × 1024.

#### 2.2.1. Temporal Convolutional Architecture

The normalized two-channel sequence X_n_ ∈ R^2 × T^, defined in Equation (2), was first projected into a higher-dimensional feature space using a pointwise one-dimensional convolution:

(4) $$H_{n}^{\left(0\right)}=\mathrm{GELU}\left(\mathrm{LN}\;\left(\mathrm{Conv}1D\left(X_{n}\right)\right)\right)$$

where LN(⋅) denotes layer normalization, and GELU denotes the Gaussian error linear unit activation function [34,35]. The pointwise convolution transforms the two normalized detector signals into C = 128 learned feature channels at each temporal position. Layer normalization stabilizes the feature statistics across channels, while GELU provides a smooth nonlinear transformation suitable for continuous phase regression.

The projected features were subsequently processed using a stack of L residual dilated convolutional blocks. The dilation factor of the l-th block was defined as

(5) $$\begin{array}{c} d_{l}=2^{l-1},\;l=1,2,\ldots ,L. \end{array}$$

For L = 4, the dilation factors were therefore d_l_ ∈ {1, 2, 4, 8}. Each residual block contained two non-causal dilated one-dimensional convolutional layers with the same dilation factor and same-length padding. Each convolution was followed by layer normalization, GELU activation, and dropout. The transformed features were then added to the block input through an identity residual connection:

(6) $$H_{n}^{\left(l\right)}=H_{n}^{\left(l-1\right)}+F_{1}(H_{n}^{\left(l-1\right)};d_{1})$$

where H_n_^(l − 1)^ ∈ R^C × T^ and $H_{n}^{\left(l\right)}\in R^{C\times T}$ denote the input and output feature maps, respectively, of the l-th residual block for the n-th sequence. The function F_l_(⋅; d_l_) represents the nonlinear residual transformation implemented by two successive Conv1D–Layer Norm–GELU–Dropout stages operating with dilation factor d_l_. Because the input and output feature dimensions were identical, the shortcut connection required no additional projection.

Since demodulation was performed offline after acquisition of the complete waveform, non-causal convolutions were used. The prediction at each temporal position could therefore incorporate information from both preceding and subsequent samples within the receptive field. This bidirectional temporal context allows the network to interpret each interferometric sample using both the nearby oscillatory structure and the broader temporal evolution of the signal, which is beneficial in the presence of measurement noise, amplitude fluctuations, and slow interferometric drift. Residual learning further improves optimization by allowing each block to refine, rather than completely replace, the features extracted by the preceding block.

The exponentially increasing dilation factors enlarge the temporal receptive field without requiring a substantially deeper network. Because each residual block contains two convolutional layers with kernel size k and dilation d_l_, effective receptive field is

(7) $$\mathrm{RF}=1+2\left(k-1\right)\sum_{l=1}^{L}d_{l}$$

For k = 3, L = 4, and d_l_ ∈ {1, 2, 4, 8}, the receptive field is 61 temporal samples. Thus, each predicted phase value is informed by a symmetric neighborhood containing 30 preceding samples, the current sample, and 30 subsequent samples. This receptive field enables the network to capture rapid local fringe variations together with slower temporal changes in the phase of the sensing signal.

After the final residual block, a pointwise one-dimensional convolution with a kernel size of one mapped the final feature representation to a single continuous dynamic-phase sequence:

(8) Δϕ^n(t)=Conv1D1 ×[HnL]1(t)=WoutHnL(t)+bout, t=1,2,…,T.

where $H_{n}^{\left(L\right)}(t)\;\in \;R^{C}$ is the final temporal feature vector of the n-th sequence at time t, and W_out_ ∈ R^1 × C^ and b_out_ ∈ R are the trainable weight and bias of the output convolution, respectively. The output therefore has dimensions 1 × T, providing one predicted dynamic-phase value for every input sample. No temporal pooling or global average pooling was applied because the temporal resolution of the input waveform must be preserved.

#### 2.2.2. Supervised Phase-Recovery Loss

The reference dynamic phase sequence, Δϕ_ref,n_(t), was generated using the conventional ellipse-fitting demodulation method. The two measured intensity signals were transformed into a pair of quadrature components using the fitted characteristic ellipse, and the corresponding continuous phase was recovered by arctangent demodulation and phase unwrapping.

The supervised phase-recovery loss was defined as

(9) $$L_{\mathrm{phase}}=\frac{1}{\mathrm{NT}}\sum_{n=1}^{N}\sum_{t=1}^{T}{[\Delta {\hat{\phi }}_{n}(t)-\;\Delta \phi_{\mathrm{ref},n}\left(t)\right]}^{2}$$

where N is the total number of sequences used in the loss calculation, and T is the number of temporal samples in each sequence. This term constrained the network to recover the magnitude, polarity, and temporal evolution of the reference phase.

#### 2.2.3. Interferometric Physics-Consistency Loss

The supervised phase-recovery loss constrains the predicted phase to follow the reference phase sequence. However, minimizing the supervised phase error alone does not ensure that the predicted phase remains consistent with the measured interferometric signals. The non-ideal interferometric forward model was therefore incorporated into the training objective as an additional physical constraint.

Because the network predicts only the dynamic phase change, the initial interferometric phase ϕ_0,n_, was added during reconstruction. The two reconstructed intensity signals were expressed as follows:

(10) $${{\hat{I}}^{\prime }}_{1,n}(t)=A_{1,n}+B_{1,n}\cos[\phi_{0,n}+\Delta {\hat{\phi }}_{n}\left(t\right)]$$

(11) $${{\hat{I}}^{\prime }}_{2,n}(t)=A_{2,n}+B_{2,n}\cos[\phi_{0,n}+\Delta {\hat{\phi }}_{n}(t)\;+\delta_{n}]$$

Here, ${\hat{I}}^{\prime }$_1_,_n_(t) and ${\hat{I}}^{\prime }$_2_,_n_(t) are the reconstructed normalized intensities for the n-th sequence. The parameters A_1_,_n_ and A_2_,_n_ are the normalized DC offsets, B_1_,_n_ and B_2_,_n_ are the normalized fringe amplitudes, ϕ_0,n_ is the initial interferometric phase, and δ_n_ is the intrinsic phase difference between two detected channels. These parameters were obtained from the calibration measurements and used only during the network training [36].

The physics-consistency loss compared the measured and the reconstructed signals:

(12) $$L_{\mathrm{physics}}=\frac{1}{2\mathrm{NT}}\sum_{n=1}^{N}\sum_{t=1}^{T}\left[{\left(\frac{{I^{\prime }}_{1,n}(t)\;-\;{{\hat{I}}^{\prime }}_{1,n}(t)}{B_{1,n}}\right)}^{2}+\;{\left(\frac{{I^{\prime }}_{2,n}(t)\;-\;{{\hat{I}}^{\prime }}_{2,n}(t)}{B_{2,n}}\right)}^{2}\right]$$

The residuals were normalized by the corresponding fringe amplitudes to prevent the channel with the larger modulation amplitude from dominating the loss. This term penalizes phase predictions that cannot reproduce the measured normalized intensity sequences through the interferometric forward model. It therefore encourages physically consistent phase recovery in the presence of channel-dependent DC offsets, unequal fringe amplitudes, detector-gain imbalance, and deviations from the nominal 2π/3 inter-channel phase difference.

#### 2.2.4. Temporal Regularization

The recovered dynamic-phase sequences are temporally continuous but may contain rapid broadband variations. To suppress isolated nonphysical fluctuations without excessively smoothing the measured response, a weak second-order temporal-regularization term was included:

(13) $$L_{\mathrm{temporal}}=\frac{1}{N(T\;-\;2)}\sum_{n=1}^{N}\sum_{t=2}^{T-1}{[\Delta {\hat{\phi }}_{n}(t\;+\;1)-\;2\Delta {\hat{\phi }}_{n}(t)\;+\;\Delta {\hat{\phi }}_{n}\left(t-\;1)\right]}^{2}$$

The second-order difference measures the local temporal curvature of the predicted phase. It penalizes isolated or rapidly changing fluctuations while producing no penalty for locally linear phase variation. The summation extends from t = 2 to t = T − 1 because both neighboring samples are required. A relatively small weighting coefficient was used to reduce nonphysical oscillations without appreciably attenuating genuine high-frequency components of the LUS response.

#### 2.2.5. Combined Physics-Guided Training Objective

The PG-TCN was trained using a weighted combination of the supervised phase-recovery, physics-consistency, and temporal-regularization losses:

L_total_ = L_phase_ + β_physics_L_physics_ + β_temporal_L_temporal_ (14)

where β_physics_ and β_temporal_ control the contributions of the physics-consistency and temporal-regularization terms, respectively. Their values were selected empirically during model development and subsequently fixed for all test measurements. Because the cosine-based interferometric response is periodic, the physics-consistency loss alone cannot uniquely determine the correct continuous-phase branch. The supervised phase target is therefore required to guide the network toward the appropriate continuous-phase solution. The physics-consistency term serves as an additional constraint that links the predicted phase to the measured intensity signals rather than as a standalone phase-recovery objective.

### 2.3. Training and Evaluation Protocol

**Figure 3:**
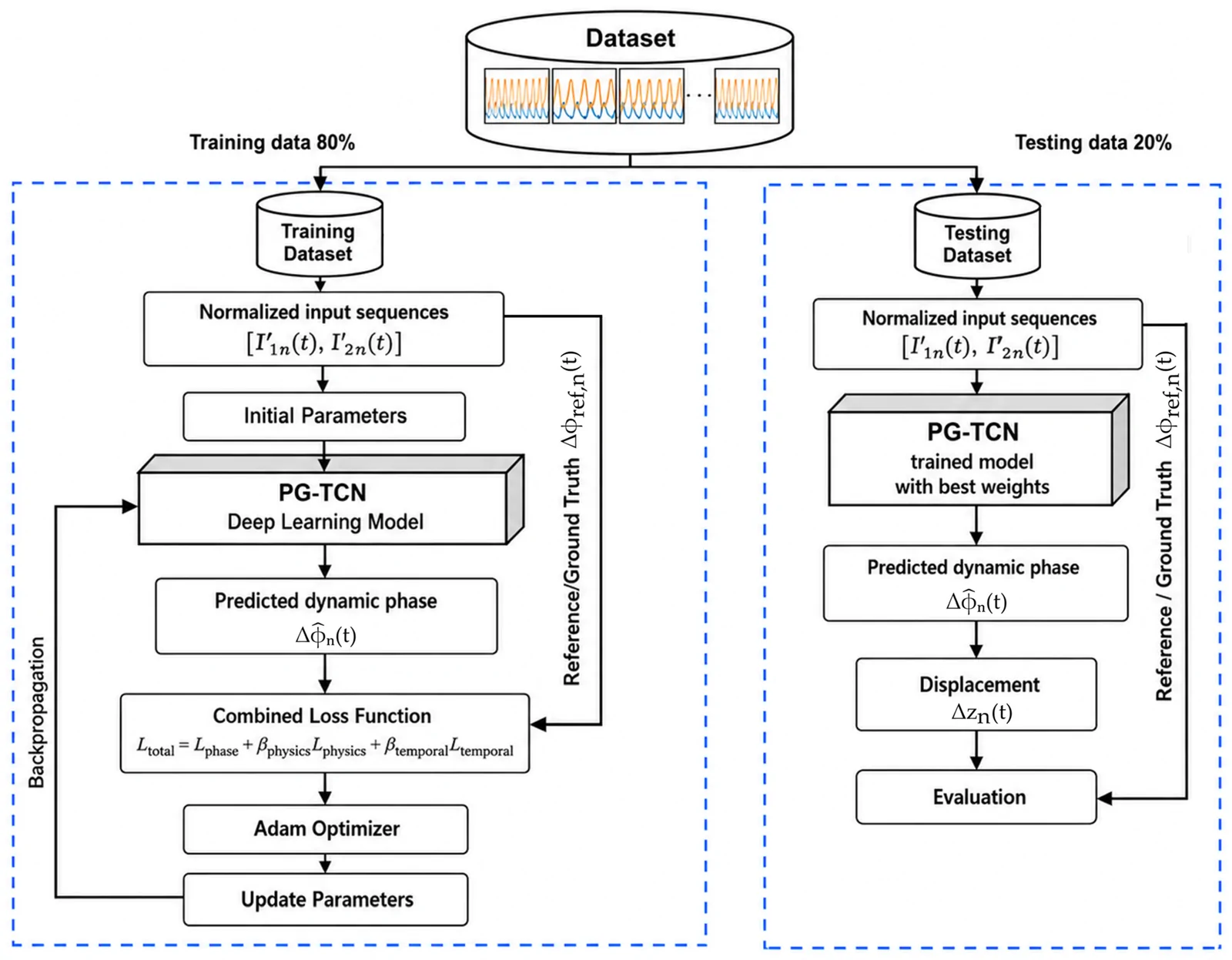
Training and testing workflow of the PG-TCN, including data normalization, physics-guided optimization with combined loss function, phase prediction, displacement recovery, and performance evaluation.

The training dataset included controlled interferometric measurements and representative LUS signals acquired under different signal conditions [22]. Each sample contained two normalized intensity sequences, I′_1_,_n_(t) and I′_2_,_n_(t), with T = 1024 temporal samples, resulting in an input dimension of 2 × 1024 and an output dimension of 1 × 1024. The overall training and evaluation workflow of the PG-TCN is illustrated in Figure 3.

The training dataset comprised 10,000 interferometric sequences pooled from multiple experiments, including PZT-modulated calibration measurements acquired under several static chamber pressures and corneal-phantom LUS measurements acquired at a chamber pressure of 15 kPa. These data were combined to train the PG-TCN. The corneal-phantom LUS measurements acquired at chamber pressures of 10 and 20 kPa were excluded from the training dataset and were evaluated only after network optimization was completed. Before training, the PZT-modulated and LUS signals were resampled to a common input length while preserving the physical duration of each signal type.

The underlying experimental platform was characterized using sinusoidal PZT modulation at 5 kHz and 25 kHz [22], with the 25 kHz condition used for small-displacement measurements. These discrete PZT conditions should not be interpreted as a continuously validated frequency range. The corneal-phantom measurements contained transient LUS responses acquired at 125 MS/s; however, their acoustic spectral bandwidth was not independently characterized. Accordingly, the present model validation is restricted to the experimentally measured PZT and LUS signal conditions represented in the dataset.

For the PZT-modulated calibration sequences, the reference dynamic phase was obtained using conventional ellipse-fitting demodulation of the measured phase-diverse signals. For the LUS sequences, the reference phase was obtained from the pooled repeated LUS acquisitions using the ellipse-based calibration procedure described in Ref. [22]. In both cases, the resulting continuous reference phase was used only for supervised training or quantitative evaluation and was not required during PG-TCN inference. The two measured intensity channels were represented as a Lissajous trajectory, and conventional ellipse fitting was used to estimate the DC offsets, fringe amplitudes, and interchannel phase separation. These parameters were then used to calculate the wrapped phase through arctangent demodulation, followed by phase unwrapping to obtain the continuous reference dynamic-phase sequence. Ellipse fitting was used only to generate the reference phase and was not included in the PG-TCN inference pipeline.

**Figure 4:**
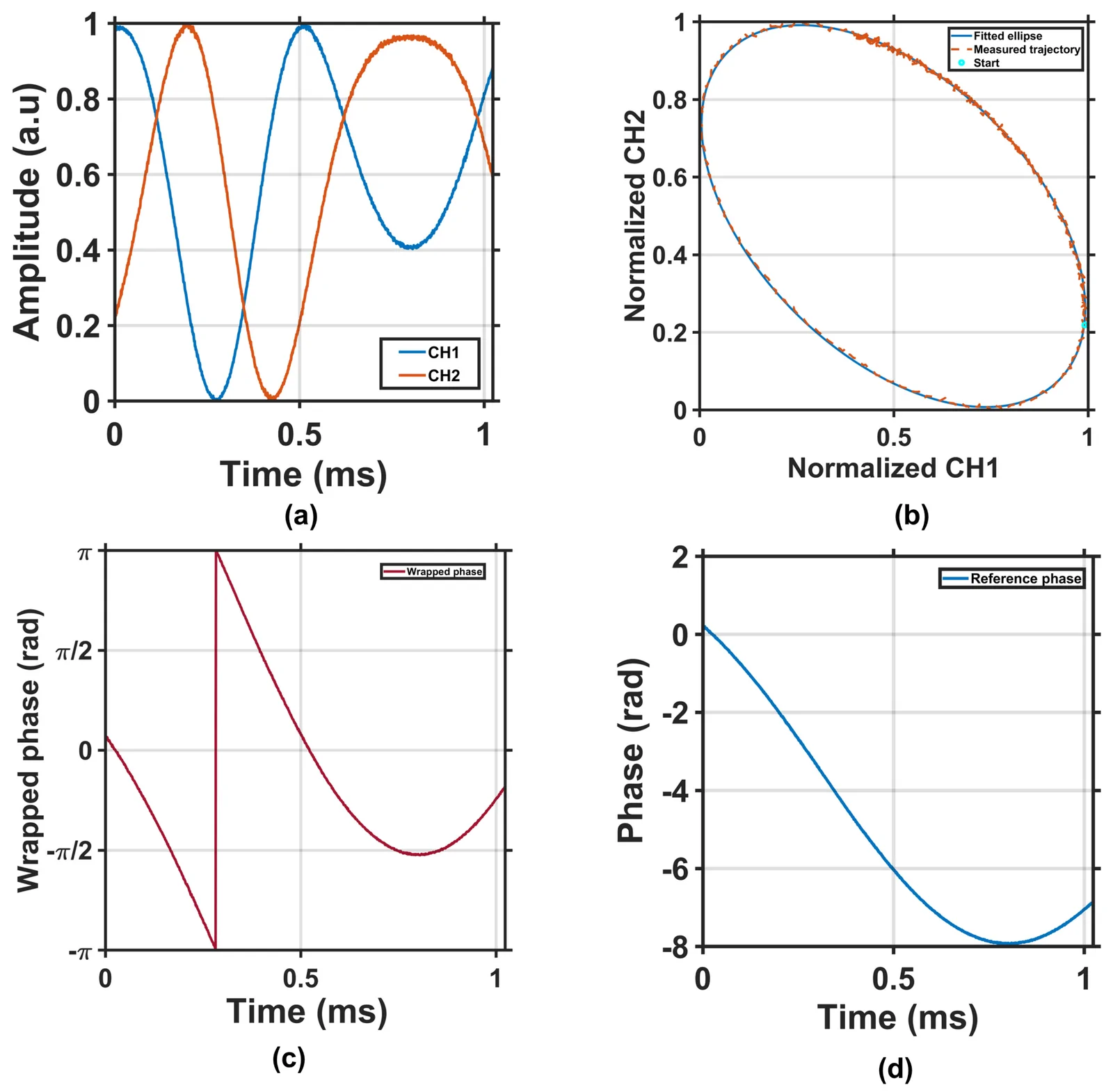
Representative training-data generation procedure. (**a**) Two normalized interferometric intensity signals, I′_1_,_n_(t) and I′_2_,_n_(t), used as the PG-TCN input. (**b**) Measured Lissajous trajectory and corresponding fitted ellipse used to estimate the interferometric parameters. The blue marker indicates the starting point of the temporal sequence. (**c**) Wrapped phase obtained through conventional ellipse-based phase demodulation. (**d**) Continuous reference dynamic phase Δϕ_ref,n_(t) obtained through conventional phase demodulation and phase unwrapping.

Figure 4 presents a representative example of the training-data generation procedure. Figure 4a shows the normalized intensity sequences, I′_1_,_n_(t) and I′_2_,_n_(t), within the range [0, 1]. The channels exhibit phase-shifted temporal variations and unequal signal excursions, reflecting the nonideal response of the practical interferometric system. In Figure 4b, the two normalized channels are represented as a Lissajous trajectory. The close agreement between the measured trajectory and the fitted ellipse indicates that the estimated interferometric parameters adequately describe the relationship between the two channels. Figure 4c shows the wrapped phase obtained from the fitted ellipse parameters, while Figure 4d shows the corresponding unwrapped continuous reference dynamic phase, Δϕ_ref,n_(t). This continuous phase sequence was used as the supervised target associated with the two-channel input sample.

During inference, only the normalized intensity sequences I′_1_,_n_(t) and I′_2_,_n_(t) were provided to the trained PG-TCN. The reference phase, calibrated forward-model parameters, and training-loss terms were not required. The predicted dynamic-phase sequence, Δ$\hat{\phi }$_n_(t), was converted into surface displacement using Equation (1). Thus, the inference pipeline operated directly on the measured normalized intensity signals.

The performance of the PG-TCN was evaluated using complementary metrics computed with respect to reference phase Δϕ_ref,n_(t). The root mean squared error (RMSE) quantified the overall prediction error:

(15) $$\mathrm{RMSE}\;=\;\sqrt{\frac{1}{\mathrm{NT}}\sum_{n=1}^{N}\sum_{t=1}^{T}{(\Delta {\hat{\phi }}_{n}(t)-\;\Delta \phi_{\mathrm{ref},n}\left(t)\right)}^{2}},$$

and the phase mean absolute error (MAE) provides a robust average error:

(16) $$\mathrm{MAE}\;=\;\frac{1}{\mathrm{NT}}\sum_{n=1}^{N}\sum_{t=1}^{T}\left|\Delta {\hat{\phi }}_{n}(t)-\;\Delta \phi_{\mathrm{ref},n}\left(t\right)\right|$$

To assess preservation of the phase waveform shape, the normalized cross-correlation (NCC) was computed:

(17) $${\mathrm{NCC}}_{n}\;=\;\frac{\sum_{t=1}^{T}(\Delta {\hat{\phi }}_{n}(t)-\bar{\Delta {\hat{\phi }}_{n}})(\Delta \phi_{\mathrm{ref},n}(t)\;-\;\bar{\Delta \phi_{\mathrm{ref},n}})}{\sqrt{\sum_{t=1}^{T}{(\Delta \hat{\phi }n(t)-\;\bar{\Delta {\hat{\phi }}_{n}})}^{2}}\sqrt{\sum_{t=1}^{T}{(\Delta \phi_{\mathrm{ref},n}(t)\;-\bar{\Delta \phi_{\mathrm{ref},n}})}^{2}}}$$

where $\bar{\Delta {\hat{\phi }}_{n}}$ and $\bar{\Delta \phi_{\mathrm{ref},n}}$ denote temporal means of the predicted and the reference phase sequences, respectively. The NCC was calculated independently for each sequence and subsequently summarized as mean ± SD for each chamber-pressure condition. Finally, the phase signal-to-error ratio (SER) quantified the strength of the reference phase relative to the residual prediction error:

(18) $$\mathrm{SER}\;=\;{10\log}_{10}\left(\frac{\sum_{n=1}^{N}\sum_{t=1}^{T}\Delta {\phi^{2}}_{\mathrm{ref},n}\left(t\right)}{\sum_{n=1}^{N}\sum_{t=1}^{T}{({\Delta \hat{\phi }}_{n}(t)-\;\Delta \phi_{\mathrm{ref},n}\left(t)\right)}^{2}}\right).$$

Together, these metrics provide a comprehensive assessment of demodulation accuracy, robustness to noise, and preservation of phase dynamics.

To further evaluate amplitude preservation during the phase demodulation, the modulation amplitudes of the interferometric inputs and the recovered phase were analyzed separately. Because the measured inputs represent optical intensity whereas the demodulated output represents optical phase, their numerical amplitudes were not directly compared. For each interferometric channel j, the peak-to-peak input modulation amplitude was calculated as

(19) $$A_{I_{j},n}=\underset{t}{\max}[I_{j,n}\left(t)\right]-\underset{t}{\min}[I_{j,n}\left(t)\right],\;j=1,2$$

The peak-to-peak dynamic-phase amplitude was calculated as

(20) $$A_{\phi ,n}=\underset{t}{\max}[\Delta \phi_{n}\left(t)\right]-\underset{t}{\min}[\Delta \phi_{n}\left(t)\right].$$

Phase-amplitude preservation was quantified using the relative amplitude error between the predicted and reference phases,

(21) $$E_{A,n}=\frac{\left|A_{\phi ,\mathrm{pred},n}-A_{\phi ,\mathrm{ref},n}\right|}{A_{\phi ,\mathrm{ref},n}}\times 100\%.$$

To evaluate sensitivity to the initial interferometric operating point, the PZT sequences were grouped into eight equally spaced intervals according to their calibrated initial phase over [–π, π]. To account for the differences in phase-excursion magnitudes, the normalized phase RMSE (NRMSE) was calculated as

(22) $${\mathrm{NRMSE}}_{n}=\frac{{\mathrm{RMSE}}_{n}}{\underset{t}{\max}[\Delta \phi_{\mathrm{ref},\;n}\left(t)\right]-\underset{t}{\min}\left[\Delta \phi_{\mathrm{ref},\;n}\left(t\right)\right]}\;\times \;100\%.$$

The NRMSE was then evaluated within each initial-phase interval. The same analysis was additionally performed separately for the measurements acquired at 10, 15, 20, and 25 kPa.

## 3. Experimental Results

### 3.1. Phase and Displacement Recovery from PZT-Modulated Measurements

The phase-demodulation performance of the proposed PG-TCN was evaluated using PZT-modulated calibration measurements acquired at four static chamber pressures: 10, 15, 20, and 25 kPa. For each chamber-pressure condition, the reference mirror was continuously displaced using a PZT, thereby producing the time-varying interferometric signals. The chamber pressure was adjusted between acquisitions and remained constant during each measurement. Changes in chamber pressure altered the optical condition of the sensing arm.

**Figure 5:**
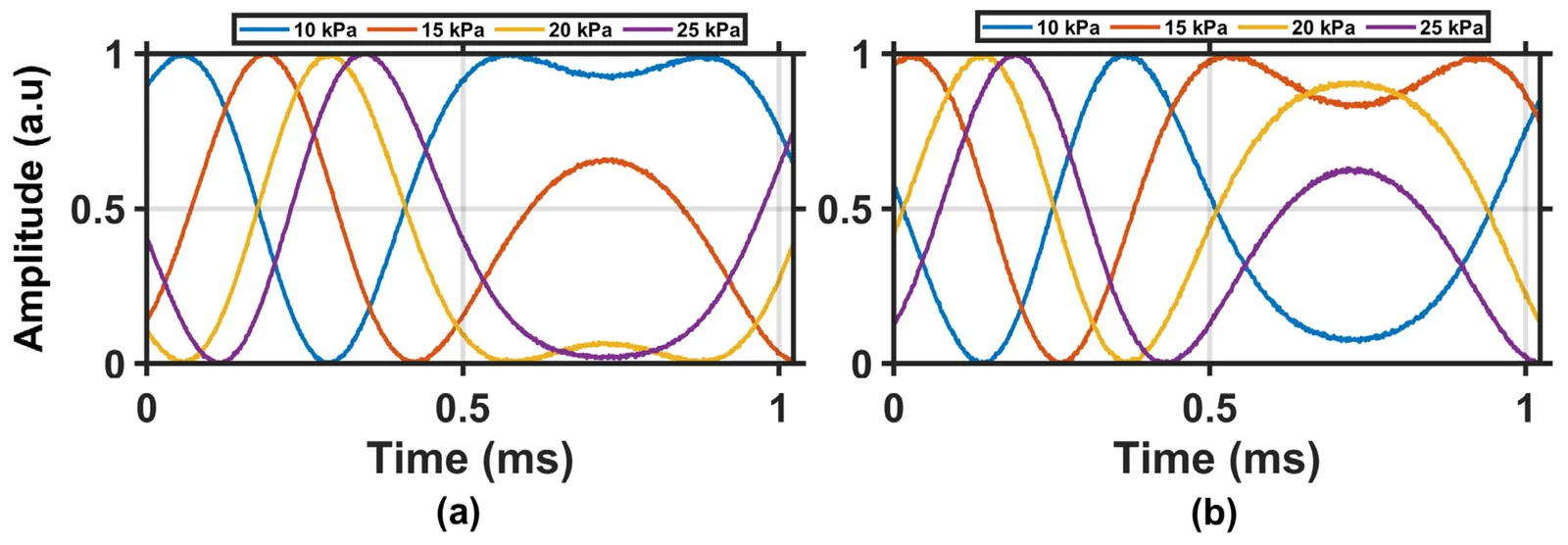
Representative normalized interferometric calibration signals generated by reference-arm PZT modulation at static chamber pressures of 10, 15, 20, and 25 kPa. Panels (**a**) and (**b**) show I′_1_,_n_(t) and I′_2_,_n_(t), respectively. The temporal variation was generated by the PZT modulation, while the pressure values represent the fixed chamber-pressure conditions.

Figure 5a and Figure 5b show representative normalized interferometric signals, I′_1_,_n_(t) and I′_2_,_n_(t), respectively, acquired at the four chamber pressures. Because the signals were normalized independently, the panels illustrate differences in waveform shape and phase evolution rather than absolute differences in fringe-modulation amplitude.

**Figure 6:**
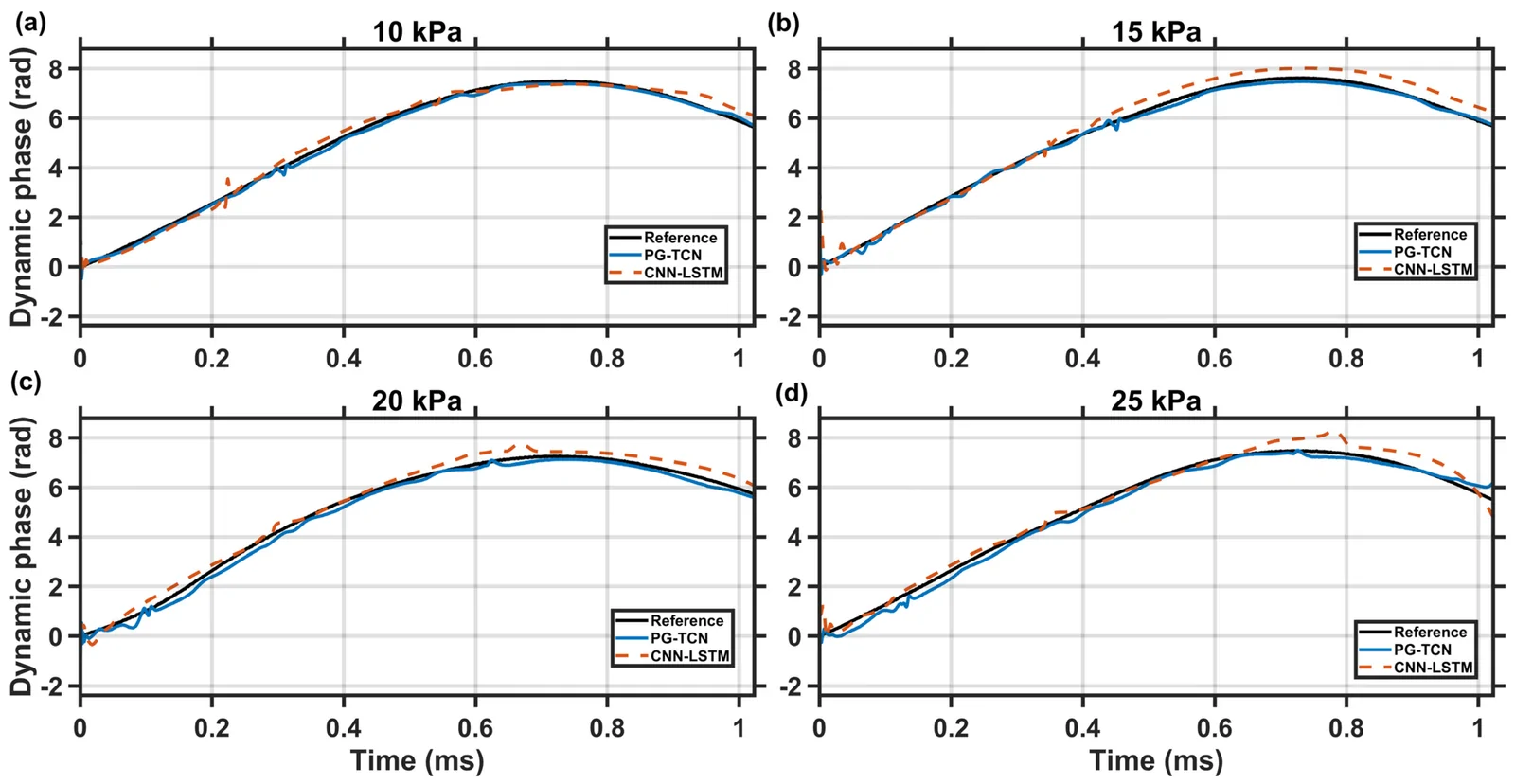
Phase-demodulation results for PZT-modulated calibration measurements acquired at static chamber pressures. Panels (**a**–**d**) correspond to 10, 15, 20, and 25 kPa, respectively. The PG-TCN and CNN-LSTM curves represent coherent averages of 30 reconstructed phase traces and are compared with the reference phase.

The phase-demodulation results are presented in Figure 6. In each panel, the dynamic-phase sequences predicted by the CNN-LSTM [33] and PG-TCN are compared with the continuous reference phase obtained using conventional ellipse-fitting demodulation. To improve the phase stability and suppress random noise, 30 reconstructed phase traces obtained under identical measurement conditions were coherently averaged for each method.

At 10 kPa, both neural demodulators reproduce the general evolution of the reference phase, although the CNN-LSTM exhibits larger localized deviations. The PG-TCN remains more closely aligned with the reference waveform. At 15 kPa, the CNN-LSTM increasingly overestimates the phase over part of the sequence, whereas the PG-TCN maintains closer agreement. At 20 kPa, both methods recover the overall phase evolution, with the PG-TCN producing a lower MAE but a slightly higher RMSE than the CNN-LSTM. At 25 kPa, the CNN-LSTM shows a more pronounced overestimation over the central and final portions of the waveform, while the PG-TCN remains comparatively close to the reference.

**Figure 7:**
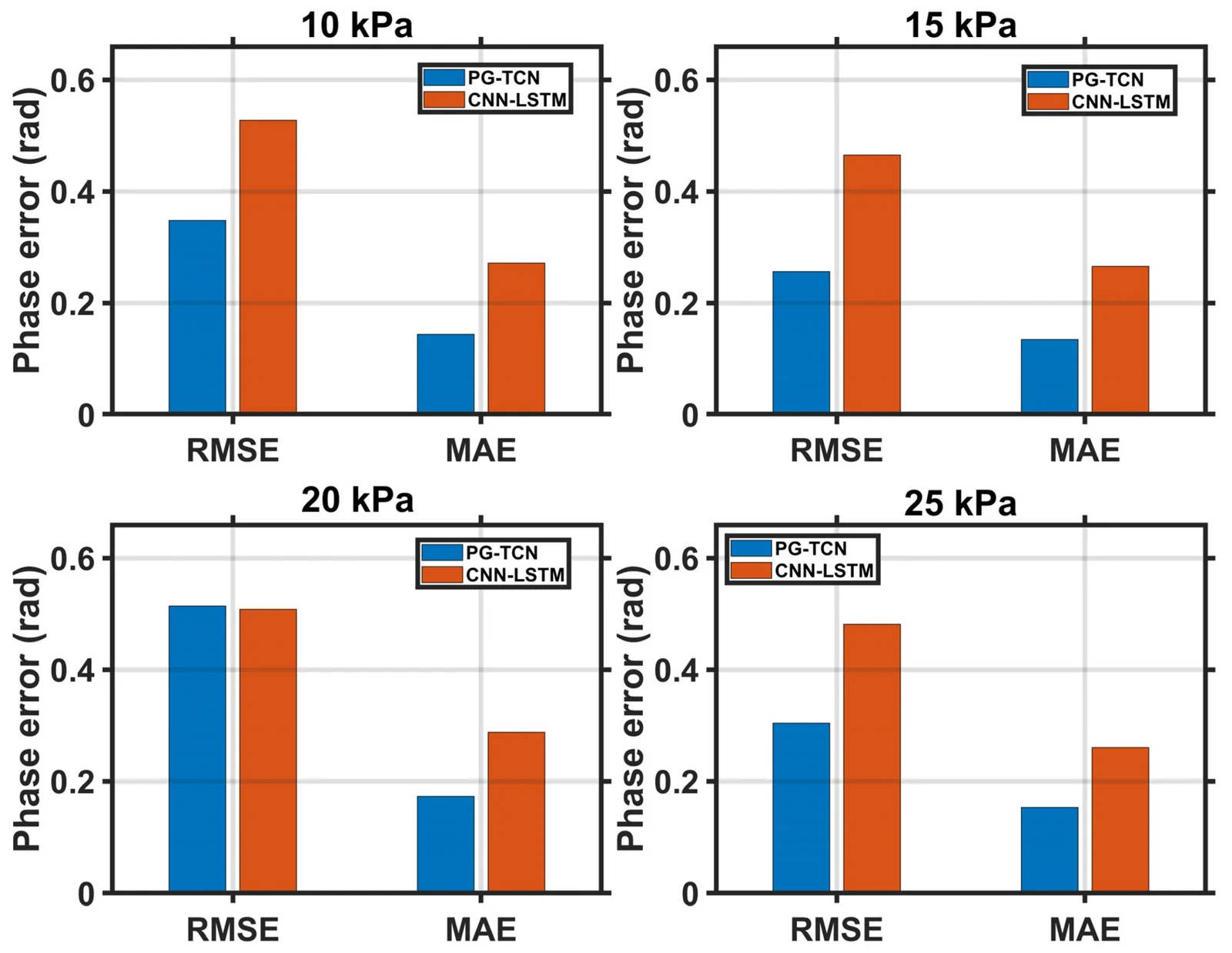
Quantitative comparison of phase-demodulation errors for PZT-modulated calibration measurements acquired at static chamber pressures of 10, 15, 20, and 25 kPa. Each panel presents the RMSE and MAE obtained using the PG-TCN and CNN-LSTM.

Figure 7 presents the phase RMSE and MAE calculated at each chamber pressure. At 10 kPa, the PG-TCN reduced the RMSE from 0.528 rad to 0.348 rad and the MAE from 0.272 rad to 0.144 rad relative to the CNN-LSTM, corresponding to reductions of approximately 34.1% and 47.0%, respectively. At 15 kPa, the corresponding reductions were 44.9% and 49.3%. At 25 kPa, the PG-TCN reduced the RMSE by 36.8% and the MAE by 41.2%.

At 20 kPa, the PG-TCN and CNN-LSTM produced similar RMSE values of 0.514 rad and 0.508 rad, respectively. However, the PG-TCN achieved a substantially lower MAE of 0.173 rad, compared with 0.288 rad for the CNN-LSTM. This combination of lower MAE and slightly higher RMSE suggests that the PG-TCN remained closer to the reference over most of the sequence, while a smaller number of comparatively large deviations had a greater influence on its RMSE.

The NCC and SER results are summarized in Table 1. The PG-TCN achieved mean NCC values between 0.9981 and 0.9988 across the four chamber pressure conditions, compared with 0.9939–0.9957 for the CNN-LSTM. The PG-TCN also produced smaller NCC standard deviations, indicating more consistent preservation of the temporal phase waveform. Its SER was higher at 10 kPa, 15 kPa, and 25 kPa. At 20 kPa, the two methods produced nearly identical SER values of 21.736 dB for the PG-TCN and 21.834 dB for the CNN-LSTM.

Overall, the PG-TCN achieved a lower phase MAE, higher NCC, and lower mean displacement RMSE across all four chamber-pressure conditions. It also produced a lower phase RMSE at three of the four conditions, with comparable performance at 20 kPa. These results indicate improved phase-recovery performance relative to the CNN-LSTM.

**Figure 8:**
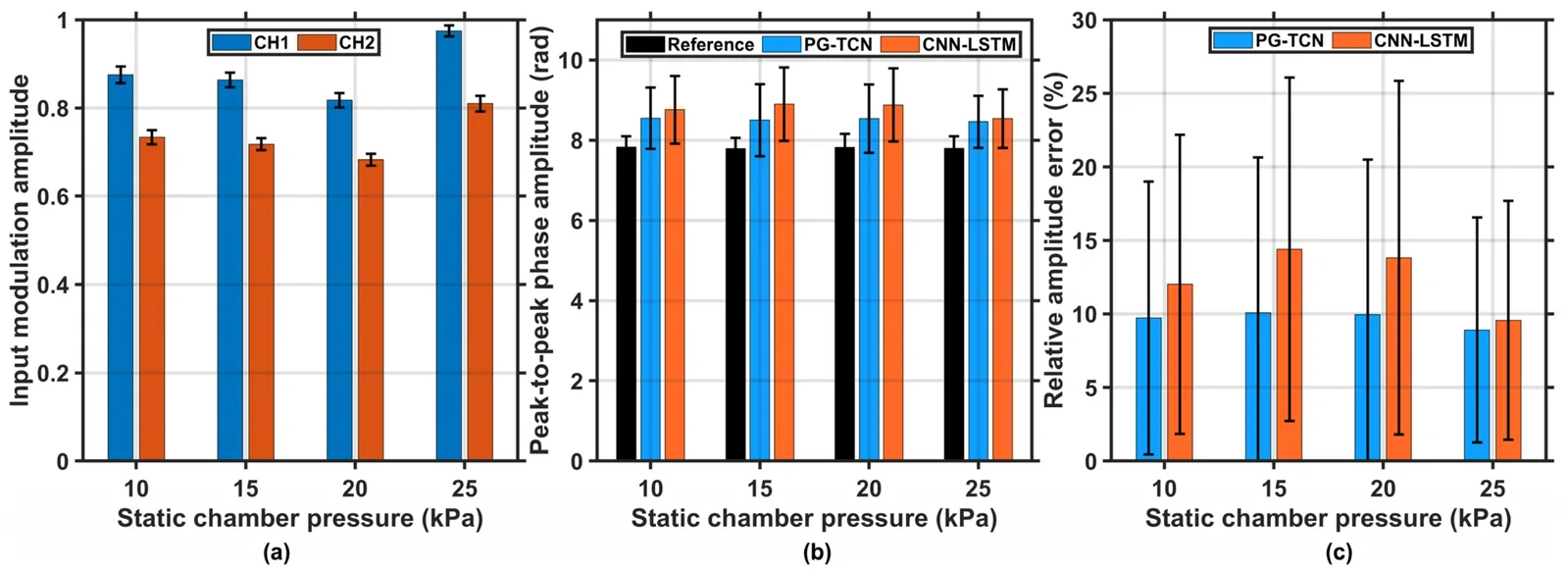
Amplitude characteristics and phase-demodulation fidelity for the PZT-modulated measurements. (**a**) Peak-to-peak modulation amplitudes of the two interferometric input channels. (**b**) Peak-to-peak dynamic-phase amplitudes obtained from the ellipse-derived reference, PG-TCN, and CNN-LSTM at chamber pressures of 10, 15, 20, and 25 kPa. (**c**) Relative phase-amplitude errors of the PG-TCN and CNN-LSTM with respect to the reference phase.

The amplitude characteristics of the two-channel demodulation were further evaluated using the PZT measurements, as shown in Figure 8. Because the interferometric inputs and the demodulated phase represent different physical quantities, their amplitudes were evaluated separately rather than directly compared. Figure 8a shows the peak-to-peak modulation amplitudes of the two interferometric channels calculated from the raw signals before normalization, thereby preserving the original intensity scale. The unequal amplitudes of the two channels reflect the channel-dependent modulation characteristics of the experimental interferometer. Figure 8b compares the peak-to-peak dynamic-phase amplitudes obtained from the ellipse-derived reference, PG-TCN, and CNN-LSTM. Both neural methods reproduced the overall modulation magnitude; however, the PG-TCN remained closer to the reference across the four evaluated chamber pressure conditions. Accordingly, Figure 8c shows that the PG-TCN produced a lower mean relative phase-amplitude error than the CNN-LSTM at each pressure level. The PG-TCN amplitude error ranged from 8.90% to 10.07%, compared with 9.56% to 14.40% for the CNN-LSTM. These results indicate improved preservation of the phase-modulation magnitude by the PG-TCN and complement the waveform-based RMSE, MAE, NCC, and SER evaluations.

Separately, the influence of the initial interferometric phase on the demodulation performance was evaluated across the PZT measurements. The measured initial phases covered nearly the complete principal interval from −π to π. The PG-TCN showed a clear dependence on the initial operating phase, with the lowest median NRMSE obtained within approximately -π/2 ≤ ϕ_0_ ≤ π/2. Within this central region, the median NRMSE ranged from 2.88% to 9.36%, whereas it increased to approximately 22–26% in the outer phase intervals near ±π. The same general trend was observed when the measurements were analyzed separately at 10, 15, 20, and 25 kPa, indicating that the observed variation was primarily associated with the interferometric operating phase rather than the chamber-pressure condition.

**Figure 9:**
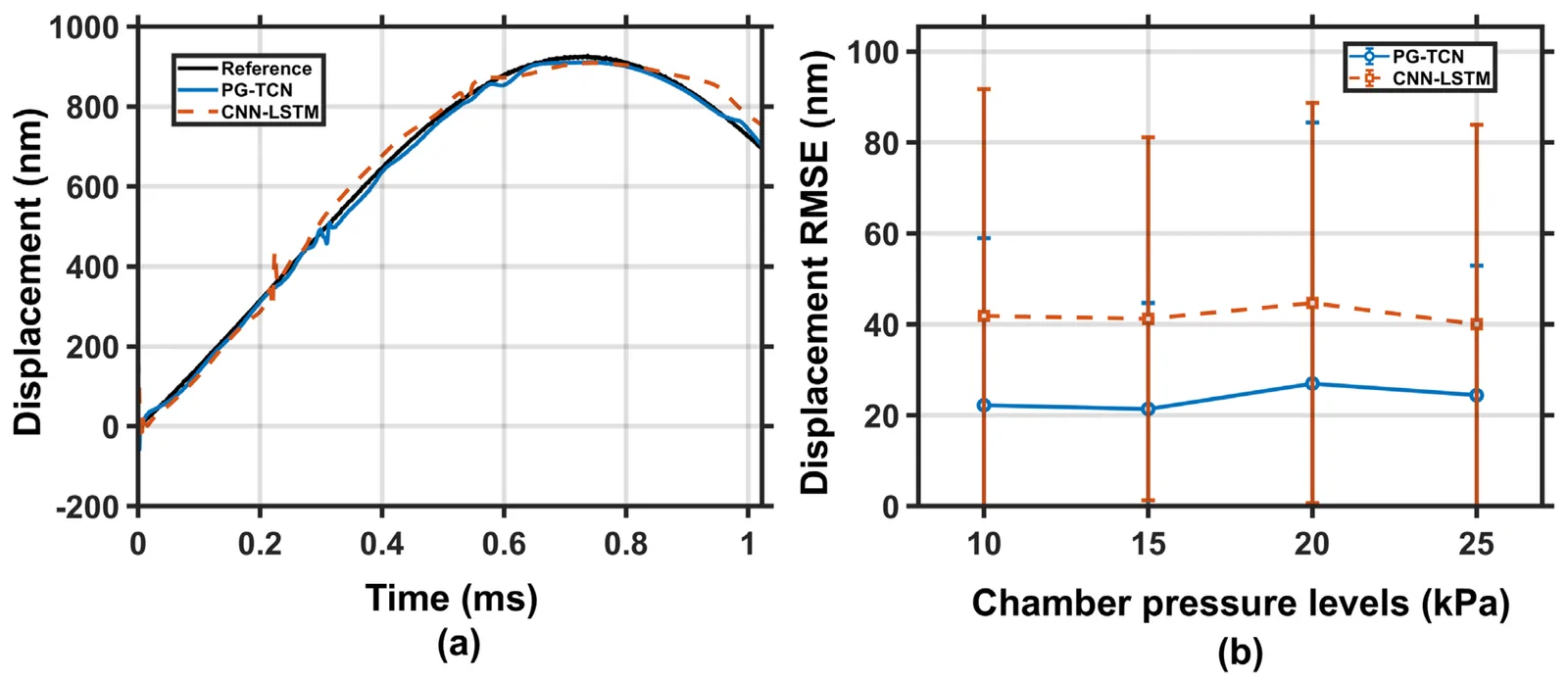
Displacement-recovery performance of the PG-TCN and CNN-LSTM for PZT-modulated calibration measurements. (**a**) Representative displacement waveforms acquired at a static chamber pressure of 10 kPa. (**b**) Mean sequence-wise displacement RMSE with standard deviation at the four chamber-pressure conditions.

The predicted dynamic phase was subsequently converted into displacement using Equation (1). Figure 9a compares representative displacement waveforms reconstructed from the reference phase and the predictions of the two neural demodulators at 10 kPa. The PG-TCN-derived displacement closely follows the reference over most of the temporal interval. The CNN-LSTM reconstruction exhibits larger deviations, particularly near rapid waveform changes and toward the end of the sequence.

Figure 9b and Table 2 summarize the sequence-wise displacement RMSE, reported as the mean and standard deviation at each chamber pressure. The PG-TCN reduced the mean RMSE from 41.828 nm to 22.219 nm at 10 kPa, from 41.212 nm to 21.407 nm at 15 kPa, from 44.687 nm to 26.995 nm at 20 kPa, and from 40.046 nm to 24.414 nm at 25 kPa. These values correspond to reductions of 46.9%, 48.1%, 39.6%, and 39.0%, respectively.

The lowest mean PG-TCN displacement error was obtained at 15 kPa, whereas the highest occurred at 20 kPa. The PG-TCN also produced lower standard deviations at 10, 15, and 25 kPa, although its variability was higher than that of the CNN-LSTM at 20 kPa. The relatively large error dispersion for both methods indicates substantial sequence-to-sequence variability; therefore, the comparison should be interpreted descriptively unless statistical significance is established using a paired test.

### 3.2. Phase and Displacement Recovery from Corneal-Phantom LUS Measurements

To evaluate the proposed photonic non-contact acoustic-detection framework in a biomedical sensing scenario, the trained PG-TCN was applied to a cornea-mimicking PDMS phantom. Corneal thickness and biomechanical response vary with intraocular pressure, motivating pressure-dependent measurements using deformable corneal phantoms [8,9]. The experimental concept followed the non-contact LUS interferometric approach reported in Ref. [22]. During these measurements, reference-arm PZT modulation was disabled, thus the time-dependent interferometric response was originated only from the LUS-induced surface displacement.

The corneal phantom consisted of a PDMS film sealed over an air chamber, forming a deformable membrane whose curvature and thickness changed with the applied chamber pressure. A transient LUS pulse was generated at the upper surface of the PDMS film. The wave propagated through the film, reflected from the lower PDMS–air interface, and returned to the upper surface, where the resulting displacement was measured using the 3 × 3 interferometer.

The present measurement should be distinguished from conventional photoacoustic microscopy of endogenous ocular absorbers. Here, carbon black was intentionally incorporated into the synthetic PDMS film to provide strong optical absorption at the surface of the sample for generating LUS pulses. The subsequent measurement therefore evaluates the acoustic propagation, reflection, and surface-displacement detection rather than spatial imaging of endogenous absorbers within ocular tissue.

The purpose of this experiment was not to perform ultrasound image reconstruction but to evaluate whether the optical interferometric detector and the neural demodulator could preserve weak transient acoustic information sufficiently for quantitative biomechanical sensing.

**Figure 10:**
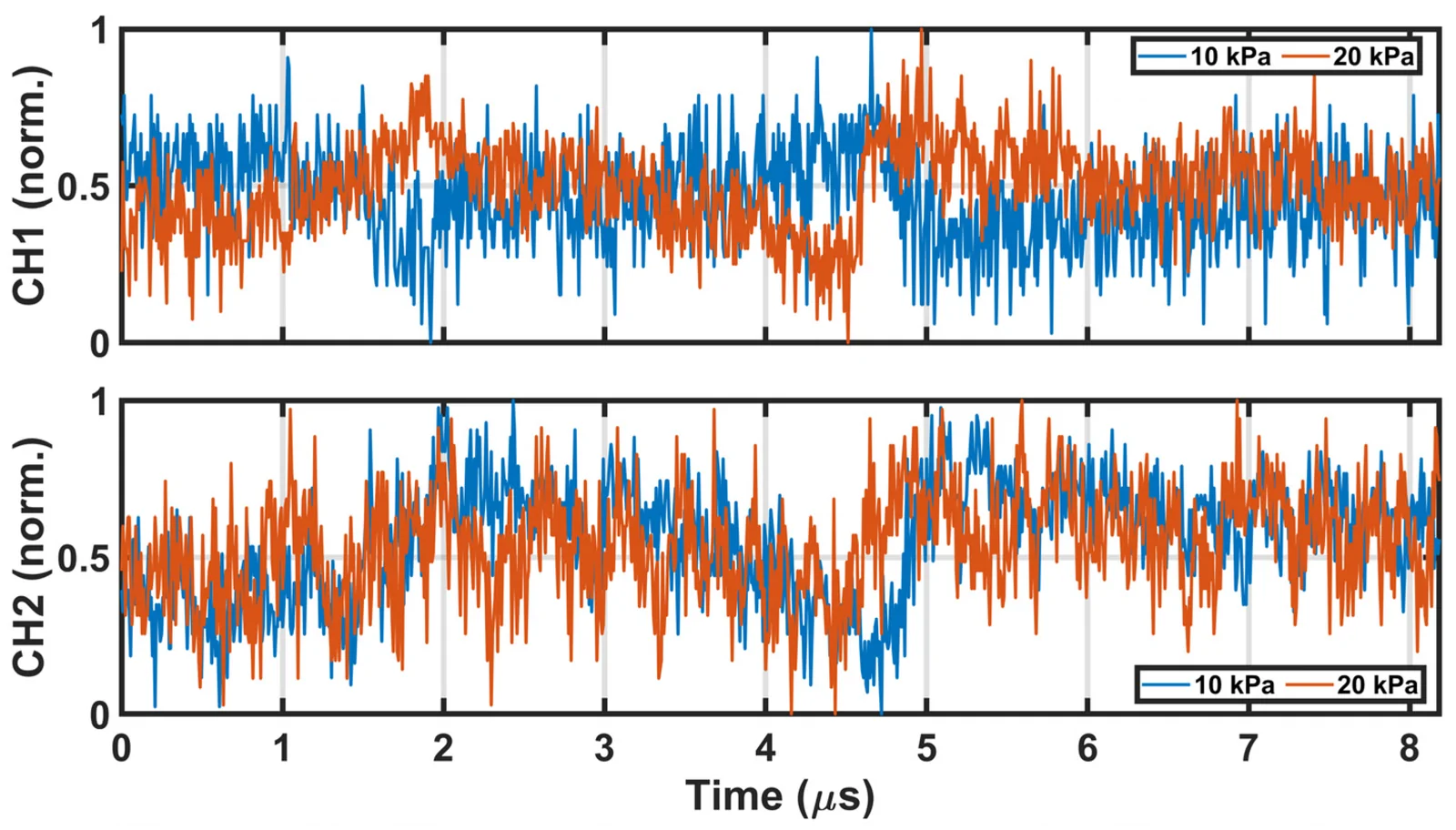
Normalized interferometric signals acquired from the corneal phantom at chamber pressures of 10 kPa and 20 kPa. The upper and lower panels show I′_1_,_n_(t) and I′_2_,_n_(t), respectively.

Measurements acquired at chamber pressures of 10 kPa and 20 kPa were considered in this study. Figure 10 shows the corresponding normalized intensity signals, I′_1_,_n_(t) and I′_2_,_n_(t). Both channels exhibit rapid interferometric variations carrying the transient LUS response. The differences between the two pressure conditions reflect pressure-dependent changes in the phantom geometry and acoustic propagation path.

**Figure 11:**
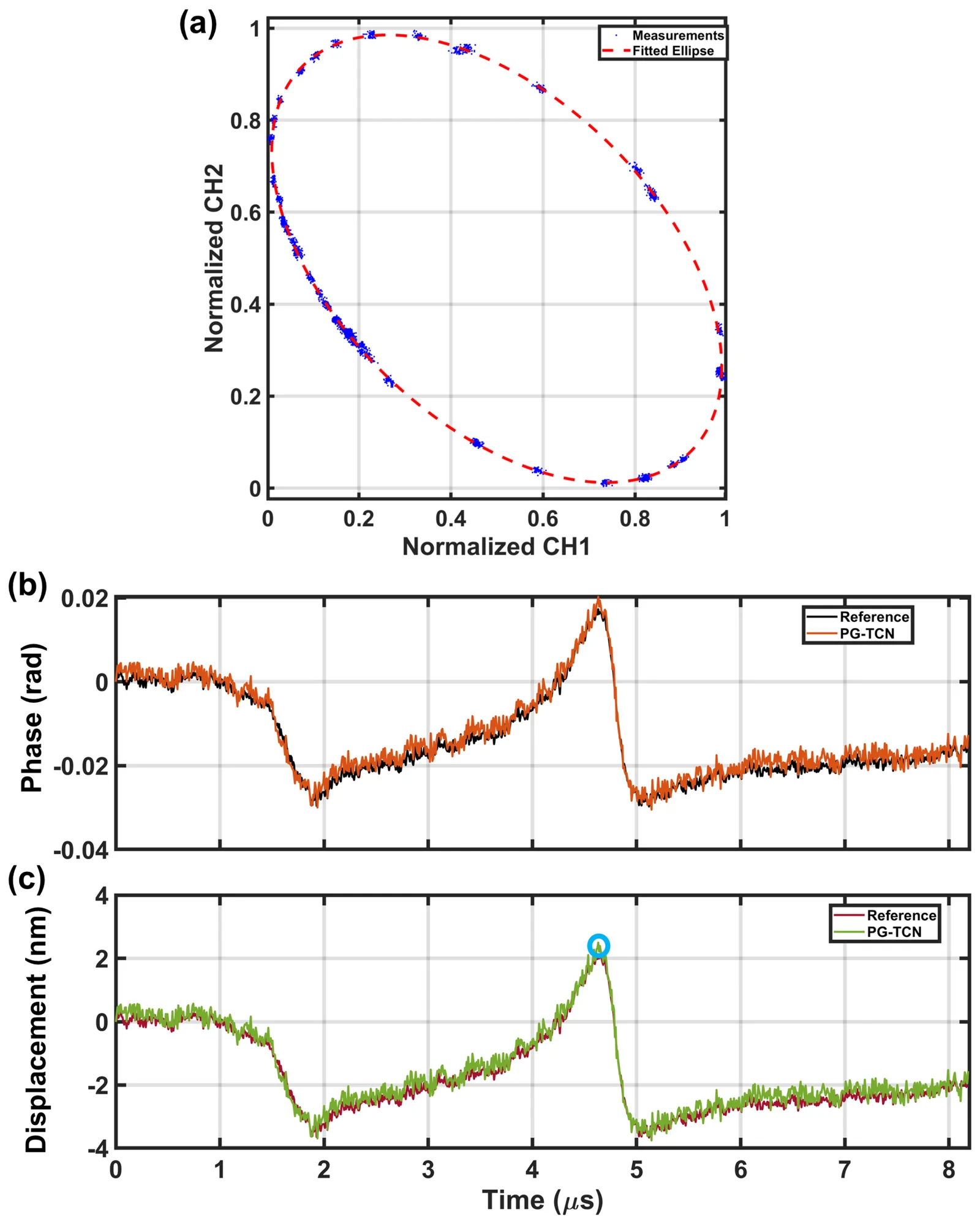
Corneal-phantom results at 10 kPa. (**a**) Normalized Lissajous trajectory and fitted ellipse; (**b**) reference and PG-TCN phase responses; and (**c**) corresponding displacement waveforms. The identified first echo arrives at 4.632 µs. Blue circle shows detected peak.

**Figure 12:**
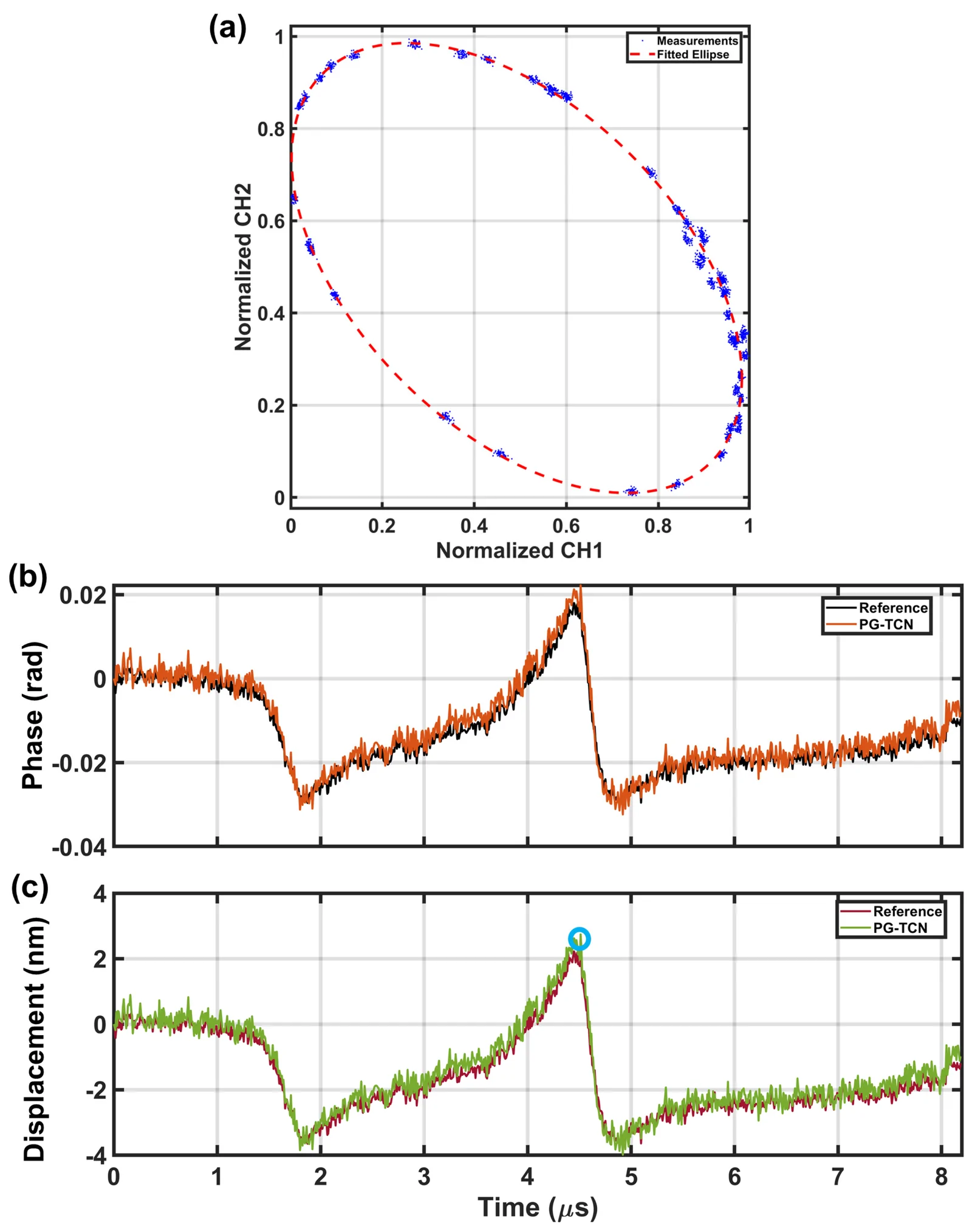
Corneal-phantom results at 20 kPa. (**a**) Normalized Lissajous trajectory and fitted ellipse; (**b**) reference and PG-TCN phase responses; and (**c**) corresponding displacement waveforms. The identified first echo arrives at 4.488 µs. Blue circle shows detected peak.

For each pressure condition, 30 repeated channel-pair measurements were demodulated with the PG-TCN, and the reconstructed phase traces were temporally aligned and coherently averaged. The ellipse-derived reference phase was used only for evaluation and was not required during PG-TCN inference. Figure 11 and Figure 12 present the results at 10 and 20 kPa, respectively.

Figure 11a and Figure 12a show the normalized Lissajous trajectories and fitted ellipses used for reference-phase generation. The pooled trajectories sample a substantial fraction of the ellipse at both pressures, providing stable reference-phase estimation. The recovered phases are compared in Figure 11b and Figure 12b. At both pressures, the PG-TCN reproduces the temporal evolution of the reference phase, including the rapid changes associated with the LUS response. The corresponding displacement waveforms are shown in Figure 11c and Figure 12c. The first echo is preserved in the PG-TCN displacement waveform, allowing its arrival time to be identified. The first-echo arrival times were 4.632 µs at 10 kPa and 4.488 µs at 20 kPa. The earlier arrival at 20 kPa indicates a shortened acoustic propagation path and, consequently, a reduction in the phantom-film thickness with increasing chamber pressure.

Because the 1024-sample window at 125 MS/s spans only 8.192 µs, which is shorter than the interval required for a second round-trip echo to return through the film, only one reliable echo was captured at each pressure. The first-echo arrival time t_p_ was identified from the coherently averaged displacement waveform as the first distinct reflected LUS echo following the excitation-associated response, with the dominant peak of this echo used as its arrival time. The same selection criterion was applied at other pressure conditions. By contrast, Ref. [22] used a longer acquisition window and resolved several successive echoes.

Because the acoustic pulse propagated from the upper surface to the lower PDMS–air interface and back, the first-echo arrival time is related to the film thickness by

(23) $$d=\frac{v\left(t_{p}-t_{0}\right)}{2}$$

where d is the film thickness, v is the acoustic velocity in PDMS, t_p_ is the measured first-echo arrival time, and t_0_ is the complete fixed timing offset between the acquisition time origin and the effective acoustic launch time at the upper phantom surface.

Operationally, t_0_ includes the fixed trigger-synchronization and acquisition delays together with other fixed propagation and detection delays between the excitation trigger and the effective acoustic time origin. The acoustic velocity in 10:1 PDMS was taken as 1076 m/s [37].

A single echo can therefore be sufficient for absolute thickness estimation when both v and the complete timing offset t_0_ are independently calibrated. In the present measurements, however, t_0_ was not independently calibrated. Absolute thickness was therefore not calculated from the single echo. Because the excitation and acquisition timing configuration remained unchanged between the 10 and 20 kPa measurements, the common timing offset cancels when the difference in first-echo arrival times is evaluated. The pressure-induced relative thickness change was consequently determined from

(24) $$∆d=d_{10}-d_{20}=\frac{v}{2}\left(t_{10}-t_{20}\right).$$

The first echo was detected at 4.632 µs at 10 kPa and 4.488 µs at 20 kPa, corresponding to arrival-time reduction of 0.144 µs. Using v = 1076 m/s, the estimated thickness reduction was approximately 78.0 µm, corresponding to thickness-reduction rate of approximately 7.8 µm/kPa over the 10 kPa pressure increase. The results are summarized in Table 3.

For comparison, Ref. [22] reported a thickness reduction of approximately 108 µm and a rate of approximately 10.8 µm/kPa. These absolute-thickness values are not reproduced here because the complete timing offset t_0_ was not independently calibrated in the present measurements. Both measurements show the same trend of decreasing film thickness with increasing chamber pressure, and the reduction rates are of the same order. The smaller rate obtained in the present study may result from differences in first-peak selection, temporal sampling resolution, signal averaging, or pressure-dependent variation in the phantom between experiments.

Overall, the PG-TCN recovered the weak transient phase and displacement responses at both chamber pressures, while the pressure-dependent shift in the first-echo arrival time enabled estimation of the relative thickness change.

### 3.3. Computational Efficiency

The computational performance of the proposed PG-TCN was compared with that of the CNN-LSTM neural demodulator and conventional ellipse-fitting demodulation. All methods were evaluated with T = 1024 temporal samples. For ellipse fitting, the measured processing time included Lissajous-trajectory fitting, phase calculation, and phase unwrapping.

Ellipse-fitting performance was evaluated using 25 individual PZT-modulated calibration sequences from each static chamber-pressure condition, resulting in 100 tested sequences. The method required an average processing time of 481 ± 22 µs per sequence. The relatively small timing variation indicates that its computational cost was primarily governed by the fixed sequence length rather than the chamber-pressure condition.

The CNN-LSTM and PG-TCN required 295 ± 30 µs and 210 ± 18 µs per sequence, respectively. Consequently, the PG-TCN reduced the processing time by 28.8% relative to the CNN-LSTM and 56.3% relative to ellipse fitting. The corresponding throughputs were approximately 4760, 3390, and 2080 sequences/s for the PG-TCN, CNN-LSTM, and ellipse-fitting methods, respectively. In addition, the PG-TCN contained 397,057 trainable parameters, representing 49.8% fewer parameters than the 790,785 parameters of the CNN-LSTM.

The computational advantage of the PG-TCN primarily arises from its fully convolutional temporal architecture. The CNN-LSTM processes temporal information recurrently, introducing sequential dependencies that restrict parallel execution. In contrast, the PG-TCN employs dilated one-dimensional convolutions with dilation factors of 1, 2, 4, and 8, allowing temporal samples to be processed in parallel while maintaining a sufficiently large temporal receptive field.

Ellipse fitting contains no trainable parameters; however, it requires explicit geometric estimation of the Lissajous trajectory, followed by phase calculation and unwrapping. Its performance may also depend on whether the measured trajectory provides sufficient ellipse coverage. Under the present PZT-modulated calibration protocol, multiple acquisitions were jointly used to obtain stable reference-phase estimates. The processing time reported in Table 4 represents the computational time per 2 × 1024 sequence and does not include the additional acquisition time required to collect repeated measurements for reference generation.

The physics-consistency and temporal-regularization terms of the PG-TCN are applied only during training and therefore introduce no additional computational burden during inference. Once trained, the PG-TCN directly maps a pair of simultaneously acquired two-channel records to a single continuous dynamic-phase sequence. Combined with the reduced detector and acquisition-channel requirements of the two-output configuration, this processing speed supports practical rapid detection of transient LUS responses.

## 4. Discussion

Robust phase demodulation remains a major challenge in 3 × 3 interferometric detections of weak transient signals. Conventional ellipse fitting depends on adequate Lissajous-trajectory coverage and may become unreliable when the phase excursion is limited or the interferometric operating condition varies, as in LUS-induced corneal deformation. The present results show that the PG-TCN can recover continuous dynamic phase from two nonideal interferometric outputs under the tested experimental conditions.

The PZT-modulated calibration measurements support the use of temporal learning combined with interferometric constraints for phase recovery. Compared with the CNN-LSTM, the PG-TCN achieved lower MAE under all static chamber-pressure conditions, higher NCC, and lower displacement RMSE, with only a marginally higher phase RMSE at one condition. The amplitude analysis further showed lower mean relative phase-amplitude errors for the PG-TCN across all four operating conditions, ranging from 8.90% to 10.07%, compared with 9.56% to 14.40% for the CNN-LSTM. The initial-phase analysis, however, showed that the PG-TCN was not completely invariant to the interferometric operating point, with higher quantitative accuracy within the central phase region and increased error toward the outer phase intervals. This trend was observed consistently when the four pressure conditions were analyzed separately, indicating that the variation was primarily associated with the interferometric operating phase rather than the chamber-pressure condition. Overall, the observed performance is consistent with the use of dilated temporal convolutions together with physics-consistency and temporal-regularization constraints during training. Because these constraints are applied only during training, they do not increase the inference cost. Moreover, both neural models reconstructed phase from a single two-channel record, whereas stable ellipse-based reference generation required multiple pooled acquisitions.

For the corneal phantom, the first detected echo shifted from 4.632 µs at 10 kPa to 4.488 µs at 20 kPa, indicating a pressure-dependent reduction in the acoustic propagation path. Based on the reported arrival-time difference and acoustic velocity of 1076 m/s, the corresponding relative thickness reduction was approximately 78 µm, or approximately 7.8 µm/kPa. This follows the same pressure-dependent trend as the approximately 10.8 µm/kPa reported previously in Ref. [22]. In the present experiment, the complete timing offset t_0_ was not independently calibrated; therefore, absolute phantom thickness was not determined from the single detected echo. A single echo would, in principle, be sufficient for absolute thickness estimation if both t_0_ and the acoustic velocity were independently known. The present analysis was therefore restricted to the relative thickness change obtained from the pressure-dependent echo shift. In addition, the phantom phase traces were obtained by coherently averaging 30 independently demodulated measurements, so fully single-shot phantom operation remains to be demonstrated.

The PG-TCN required only 210 ± 18 µs per sequence, compared with 295 ± 30 µs for the CNN-LSTM and 481 ± 22 µs for ellipse fitting, while using approximately half the trainable parameters of the CNN-LSTM. These results indicate a computational advantage for rapid processing of transient interferometric measurements under the present implementation.

From a biosensing perspective, the main contribution of this work lies in the optical detection of weak acoustic responses rather than photoacoustic imaging. The corneal phantom should likewise be interpreted as a pressure-responsive mechanical and acoustic test structure rather than a complete model of corneal anatomy or optical absorption. The carbon-black-mediated surface excitation used here differs from the conventional photoacoustic imaging based on endogenous ocular chromophores. The 3 × 3 fiber interferometer acts as a non-contact acoustic sensor, while the PG-TCN converts its nonideal optical outputs into quantitative optical phase and surface displacement. From a practical implementation perspective, using two phase-diverse outputs reduces the required detector and digitizer-channel counts while retaining the information required for the present neural phase-demodulation strategy. The same general detection concept may therefore also be relevant to photoacoustic systems employing interferometric optical ultrasound detection [38], although such applications will require dedicated validation.

Several limitations remain. Only two phantom pressures were evaluated, a constant acoustic velocity was assumed, the phantom responses required coherent averaging, and an ellipse-derived reference phase was used for supervision. The present implementation also uses only two of the three available 3 × 3-coupler outputs; inclusion of the third output could provide additional redundancy under conditions such as low optical power, channel imbalance, or severe noise, but this remains to be experimentally assessed. Optical source power and fiber transmission loss were not independently varied. Although channel-wise normalization reduces sensitivity to purely multiplicative intensity variations, reduced received optical power can decrease the interferometric signal-to-noise ratio and thereby degrade demodulation accuracy. Controlled measurements over different received optical-power and attenuation levels are therefore required to quantify this dependence.

The sequence-wise min–max normalization introduces an additional limitation because its scaling is determined by the extrema of each individual record. Isolated outliers can therefore affect the normalized range, particularly for weak signals with small phase excursions, and independent normalization removes the original absolute modulation-amplitude information from the neural-network input. In the present study, absolute input modulation was consequently evaluated separately using the raw pre-normalization interferometric signals. Calibration-based fixed scaling or robust percentile-based normalization may reduce sensitivity to outliers while retaining more information about absolute modulation strength. In addition, the acoustic bandwidth of the measured LUS responses was not independently characterized. Although the 125 MS/s digitization rate corresponds to a Nyquist frequency of 62.5 MHz, this represents only the digital sampling limit and not the experimentally established bandwidth of the interferometric detector or PG-TCN. The present validation should therefore not be extrapolated directly to substantially higher-frequency photoacoustic signals. Further work should evaluate broadband operation, single-shot measurements, a wider pressure range, independently calibrated phantom thickness and acoustic velocity, robustness to interferometer drift, broader operating-point diversity during training, and uncertainty-aware echo detection before extension to ex vivo and in vivo corneal measurements.

## 5. Conclusions

We have presented a physics-guided temporal convolutional network for direct phase demodulation in a non-contact 3 × 3 fiber-optic acoustic detection system. The PG-TCN recovered the continuous dynamic input phase without ellipse fitting or separate phase unwrapping during inference. It generally outperformed the CNN-LSTM on the PZT-modulated calibration measurements, achieving lower MAE, higher NCC, and lower displacement RMSE across the tested static chamber-pressure conditions. It also reduced the processing time to 210 ± 18 µs per sequence while using approximately half the trainable parameters of the CNN-LSTM.

The method was further applied to weak LUS signals from a corneal phantom at chamber pressures of 10 and 20 kPa. The detected first-echo shift corresponded to a relative thickness reduction of approximately 78 µm, or 7.8 µm/kPa, consistent with the expected pressure-dependent trend. These findings demonstrate the potential of PG-TCN for rapid and non-contact interferometric sensing of transient displacement. Future work will focus on single-shot phantom measurements, broader pressure ranges, independently calibrated acoustic properties, and validation using biological corneal samples. These findings support the use of physics-guided 3 × 3 interferometry as a rapid photonic detection approach for weak transient acoustic signals in laser-ultrasound and photoacoustic biosensing.

## Figures and Tables

**Table 1:** NCC and SER of the phase-demodulation methods at different chamber pressure levels.

Pressure Level	Method	NCC, Mean ± SD	SER (dB)
10 kPa	PG-TCN	0.99878 ± 0.00150	25.071
	CNN-LSTM	0.99528 ± 0.01119	21.447
15 kPa	PG-TCN	0.99877 ± 0.00083	27.863
	CNN-LSTM	0.99571 ± 0.00415	22.691
20 kPa	PG-TCN	0.99805 ± 0.00385	21.736
	CNN-LSTM	0.99530 ± 0.00454	21.834
25 kPa	PG-TCN	0.99816 ± 0.00119	25.959
	CNN-LSTM	0.99391 ± 0.02199	21.976

**Table 2:** Displacement RMSE for PZT-modulated calibration measurements acquired at different static chamber pressures.

Pressure Level	PG-TCN RMSE (nm), Mean ± SD	CNN-LSTM RMSE (nm), Mean ± SD
10 kPa	22.219 ± 36.699	41.828 ± 49.901
15 kPa	21.407 ± 23.294	41.212 ± 39.929
20 kPa	26.995 ± 57.401	44.687 ± 44.003
25 kPa	24.414 ± 28.516	40.046 ± 43.839

**Table 3:** Corneal-phantom thickness change estimated from LUS first-echo arrival times.

Quantity	Value
First-echo arrival time at 10 kPa, t_10_	4.632 µs
First-echo arrival time at 20 kPa, t_20_	4.488 µs
Arrival-time reduction, t_10_ − t_20_	0.145 µs
Longitudinal velocity in PDMS [37]	1076 m/s
Estimated thickness reduction, Δd (10 → 20 kPa)	78.0 µm
Thickness-reduction rate	7.8 µm/kPa
Reference rate, Ref. [22]	10.8 µm/kPa

**Table 4:** Computational performance of ellipse fitting, CNN-LSTM, and PG-TCN for an input sequence of 2 × 1024.

Method	Trainable Parameters	Processing Time (µs/Sequence), Mean ± SD	Throughput (Sequences/s)
Ellipse fitting	-	481 ± 22	2080
CNN-LSTM	790,785	295 ± 30	3390
PG-TCN	397,057	210 ± 18	4760

## Data Availability

The data presented in this study are available on request from the corresponding author.
